# Effects of betel nut on cardiovascular risk factors in a rat model

**DOI:** 10.1186/1471-2261-12-94

**Published:** 2012-10-24

**Authors:** Mohammad Perwaiz Iqbal, Naseema Mehboobali, Ghulam Haider, Shahid Pervez, Iqbal Azam

**Affiliations:** 1 Departments of Biological & Biomedical Sciences, Aga Khan University, Stadium Road, Karachi, 74800, Pakistan; 2 Pathology and Microbiology, Aga Khan University, Stadium Road, Karachi, 74800, Pakistan; 3 Community Health Sciences, Aga Khan University, Stadium Road, Karachi, 74800, Pakistan

**Keywords:** Areca nut, Betel nut, Cardiovascular disease, Metabolic syndrome, Rat model

## Abstract

**Background:**

Areca nut (commonly known as betel nut) chewing has been shown to be associated with metabolic syndrome and cardiovascular disease (CVD). The mechanism by which betel nut ingestion could lead to development of CVD is not precisely known; however, dyslipidemia, hyperhomocysteinemia, hypertriglyceridemia and inflammation could be some of the potential risk factors. This study was undertaken to investigate the effects of two dosages of betel nut on homocysteinemia, inflammation and some of the components of metabolic syndrome, such as hypertriglyceridemia, low HDL-cholesterol, obesity and fasting hyperglycemia in a rat model.

**Methods:**

Thirty-six adult female Sprague Dawley rats, aged 10–12 weeks were divided into three equal groups. Group-1 served as the control group (n = 12) and received water, whereas groups 2 and 3 were given water suspension of betel nut orally in two dosages, 30 mg and 60 mg, respectively for a period of 5 weeks. At the end of the fifth week, the animals were weighed and sacrificed, blood was collected and liver, kidney, spleen and stomach were removed for histological examination.

Plasma/serum was analyzed for glucose, total cholesterol, HDL-cholesterol, LDL-cholesterol, triglycerides, homocysteine, folate, vitamin B_12_ and N-acetyl-β-D-glucosaminidase (NAG) – a marker of inflammation.

**Results:**

When the mean concentration values of 3 groups were compared using one way ANOVA followed by Tukey’s HSD-test, there was a significant increase in the concentration of total cholesterol (p = 0.04) in the group receiving 30 mg/day betel nut compared to the control group. However, administration of a higher dose of betel nut (60 mg/day) had no significant effect on the serum concentrations of glucose, total cholesterol, HDL-cholesterol, LDL-cholesterol, and NAG. Histological examination of spleen revealed a dose-dependent extramedullary hematopoiesis. No other remarkable change in the tissues (liver, kidney and stomach) was observed.

Mean serum/plasma levels of folate, vitamin B_12_ and homocysteine were not found to be significantly different in all the groups. Betel nut ingestion had no effect on the mean body weights of rats.

**Conclusions:**

Low dosage of betel nut is found to be associated with hypercholesterolemia. However, betel nut ingestion is not associated with hyperhomocysteinemia, hypertriglyceridemia, hyperglycemia, inflammation and increase in body weight in a rat model.

## Background

Areca nut (commonly known as betel nut) is the fourth most commonly used addictive substance after caffeine, nicotine and alcohol [1]. It is estimated that nearly 600 million people engage in betel nut chewing for stress reduction, feeling of well-being and alertness etc. [2]. However, its habitual use has significant adverse effects, such as cancer of oral cavity, esophagus, stomach, liver, prostate, cervix and lungs [3]. Areca nut chewing has also been shown to increase the risk of many chronic non-communicable diseases, such as obesity, type 2 diabetes mellitus, hypertension, hyperlipidemia, metabolic syndrome and cardiovascular disease [4-8]. The mechanism by which betel nut ingestion could lead to the development of cardiovascular disease (CVD) is not precisely known, however, folate and vitamin B_12_ deficiencies leading to hyperhomocysteinemia [9], obesity [10], hypertriglyceridemia [7], malabsorption of nutrients [11], activation of sympathetic nervous system leading to impaired baroreflex buffering [12] could be some of the potential risk factors. Most of the work on the relationship between CVD and betel nut chewing has been through population based cohort studies where risk factors were assessed primarily on the basis of use and non-use of betel nut. Very few studies have investigated the effects of two different doses of betel nut in relation to cardinal risk factors for CVD. The present study was undertaken to investigate the effects of two different dosages of betel nut on homocysteinemia, inflammation and some of the components of metabolic syndrome, such as hypertriglyceridemia, low HDL-cholesterol, obesity and fasting hyperglycemia in a rat model.

## Methods

### Animals

In this animal study, 36 adult female Sprague Dawley rats, aged 10–12 weeks with weights ranging from 162–194 gm were experimented upon for a period of 35 days (5 weeks). They were housed in the animal facility at the Aga Khan University. The rats were allowed to have free access to food and clean water under conditions of 12- hour dark and light cycle and a temperature ranging from 20-23°C. Food used was the standard diet for rodents in pellet form. Rats were observed for signs of abnormalities throughout the study. They were kept untreated for 2 days for complete acclimation to the laboratory conditions. The animals were divided into 3 groups, each group comprising of 12 animals. The group 1 served as the control group (n = 12) and received water, whereas groups 2 and 3 were given water suspension of betel nut (orally) every day in the morning in dosages 30 mg and 60 mg, respectively for a period of 5 weeks.

### Drug treatment

For the rats in the two treatment groups, we selected 30 mg per day and 60 mg per day dosages of betel nut. These dosages were based on the fact that the classical doses of betel nut in humans have been reported to be from 2–8 gm for various clinical conditions [13]. Assuming an average human body weight of 55 kg, the highest dose would be 145 mg/kg. Considering that the mean body weight of rats during the study period remains around 200 gm, the average dose of betel nut would be approximately 30 mg per day. Moreover, it has been shown that betel nut meal consumption in concentrations from 5-10% by albino rats for a period of 4 weeks produced no detectable pathological changes [14]. On the basis of these two lines of evidence, betel nut doses of 30 mg and 60 mg were selected for the two treatment groups (group 2 and group 3) respectively.

### Preparation of betel nut suspension

Dried raw areca nut seeds were obtained from the whole-sale market in Karachi to ensure that the material in this study represents the main ingredient of various forms in which it is usually consumed by the general population. Dried nuts were ground into a powder form using an electric grinder. Water suspensions of 30 mg/mL and 60 mg/mL were prepared and placed on a Red Rocker [Hoefer Scientific Instruments, San Francisco] for 30 minutes for gentle mixing. Using a 3-mL syringe and an oral gavage needle, 1 mL of betel nut suspension was delivered in the lower end of the buccal cavity of the rat.

### Treatment period

One of the objectives of the study was to investigate the effect of betel nut on weight gain in adolescent rats. Since the maximum weight gain in adolescent female Sprague Dawley rats is up to a period of 4–5 weeks [15], it was decided that the treatment with betel nut would be carried out for a period of 5 weeks, starting at the age of 10–12 weeks. Moreover, a preliminary experiment was also run which indicated that the maximum growth of adult female Sprague Dawley rats was from a mean age of 10 weeks to 16 weeks under the conditions of the Animal Care facility of our University. Rats were weighed in each group every week to determine any change in body weight.

### Scarification and collection of blood and tissues

At the end of 5 weeks of treatment, the animals were weighed and anesthetized after being subjected to fasting for 12 hours. Blood (3–4 mL) was collected from the heart of each rat by cardiac puncture and transferred in equal volumes to an EDTA tube and another tube without any anti-coagulant. The rats were sacrificed by cervical dislocation while they were under the influence of anesthesia [16]. Liver, kidney, spleen and stomach were removed from each rat and fixed in 10% buffered formalin for 24 hours. Plasma and serum were separated immediately and stored at -20^0^C until analysis. The study had been approved by the Ethics Committee for Research on Animals, Aga Khan University.

### Measurements of biochemical parameters

Kits for fasting serum glucose, total cholesterol, high density lipoprotein (HDL)-cholesterol, low density lipoprotein (LDL)-cholesterol and triglycerides were obtained from Roche Diagnostics, USA. Serum sample (200 μL) was analyzed on Cobas C III analyzer (Roche Diagnostics, USA). The minimum limits of measurement for serum glucose, total cholesterol, HDL-cholesterol, LDL-cholesterol and triglycerides were 2.0 mg/dL, 9.7 mg/dL, 3.0 mg/dL, 3.9 mg/dL and 8.9 mg/dL, respectively.

Serum folate and vitamin B12 were analyzed by radioassays [17,18]. Both of these methods have been described in detail in a recent publication [19]. Determination of plasma homocysteine was carried out using a kit based on fluorescence polarization immunoassay (Abbott Laboratories, Ltd, Pakistan) following the instructions of the manufacturer. The limit of detection of homocysteine by this assay is 4 μmol/L. N-acetyl-beta-D-glucosaminidase (NAG)-a nonspecific marker of inflammation was determined in serum samples using a spectrofluorimetry based method of Whiting et al. [20]. One unit of NAG activity was defined as that which released 1 μmole of 4-methylumbelliferone per minute under assay conditions. This method has been described in detail in a previous communication [21]. Serum alanine aminotransferase (ALT) was analyzed spectrometrically using a kit (RANDOX, UK) following the manufacturer’s instructions. The lower detection limit of this assay is 8.0 units/L.

### Morphological studies

Liver, kidney, spleen and stomach immersed in 10% buffered formalin for 24 hours were processed in a routine fashion and embedded in paraffin. Serial sections (3-5μm) were cut and stained with Hematoxylin and Eosin (H & E). Slides were then examined under light microscope by a histopathologist for any morphological changes. Extramedullary erythropoiesis in splenic sections of rats from all three groups was done in a semi quantitative method. Foci of extramedullary erythropoiesis were measured in millimeter using microstage and ocular scale on Olympus BX51 microscope. The sums of all foci showing extramedullary erythropoiesis were then compared in three groups.

### Statistical analysis

Descriptive results pertaining to the experimental groups are reported as mean ± SD for body weight, lipid profile, serum glucose, serum ALT, serum NAG , serum folate, serum vitamin B_12_ and plasma homocysteine. Levels of lipids, glucose, ALT, NAG, folate, vitamin B_12_ and homocysteine in the three groups of rats were analyzed using one way ANOVA because the assumptions of normality and equality of variance were met. Significant association by groups was then followed by Tukey’s HSD test for multiple pair-wise comparisons. The differences in mean body weights of rats over a period of 5 weeks by experimental groups were compared using repeated measures ANOVA. A p-value < 0.05 was considered significant. Post-hoc power of the study was determined using PASS 2008 software (NCSS, LLC 329 North 1000 East, Kaysville, Utah 84037, USA).

## Results

Table 1 shows mean serum levels of total cholesterol, LDL-cholesterol, HDL-cholesterol, triglycerides, serum glucose, ALT and NAG in rats given betel nut suspension in water in dosages 30 mg and 60 mg per day. Only total cholesterol concentration (p = 0.04) was found to be significantly associated with the experimental groups using one way ANOVA. The pair-wise comparison showed a significant increase in the concentration of total cholesterol (p = 0.04) in the group receiving 30 mg/day betel nut compared to the control group. However, administration of a higher dose of betel nut suspension did not produce any significant effect on the levels of serum glucose, serum HDL-cholesterol, LDL-cholesterol, ALT and NAG compared to the control group. Similarly, no significant associations in the mean serum concentrations of folate, vitamin B_12_ and plasma homocysteine for the three groups were observed [Table 2].

**Table 1:** Effect of betel nut on lipid profile, serum glucose, alanine aminotransferase and N-acetyl-β-D-glucosaminidase

***Variable***	***Group 1 (Control)***	***Group 2 (30 mg betel nut/day)***	***Group 3 (60 mg betel nut/day)***	***P-value***
	***n = 12***	***n = 12***	***n = 12***	
**Mean ± SD**				
***Total cholesterol (mg/dL)***	70.7 ± 15.3	92.4 ± 23.0*	80 ± 21.7	0.04
***LDL-cholesterol (mg/dL)***	11.6 ± 4.9	14.3 ± 6.0	11.7 ± 6.0	0.43
***HDL-cholesterol (mg/dL)***	60.0 ± 11.9	73.3 ± 15.7	66.7 ± 16.4	0.11
***Triglycerides (mg/dL)***	56.3 ± 14.0	82.3 ± 44.8	60 ± 36.6	0.14
***Glucose (mg/dL)***	92.7 ± 30.5	85.7 ± 36.0	82.6 ± 24.8	0.72
***ALT (U/L)***	63.0 ± 25.7	56.0 ±19.2	42.0 ± 15.4	0.10
***NAG (U/L)***	7.0 ± 1.0	7.0 ± 1.7	7.0 ± 1.0	0.99

ALT = Alanine aminotransferase.

NAG = N-acetyl-β-D-glucosaminidase.

*p = 0.04; one way ANOVA followed by Tukey’s HSD test were used to compare mean values.

**Table 2:** **Effect of betel nut on serum folate, serum vitamin B**_**12 **_**and plasma homocysteine**

***Variable***	***Group 1 (Control)***	***Group 2 (30 mg betel nut/day)***	***Group 3 (60 mg betel nut/day)***	***P-value***
	***n = 12***	***n = 12***	***n = 12***	
**Mean ± SD**				
***Serum folate (ng/mL)***	26.6 ± 9.6	32.0 ± 11.8	27.7 ± 8.9	0.42
***Serum vitamin B***_***12***_***(pg/mL)***	284.0 ± 93.5	305.0 ± 76.8	295.8 ± 98.0	0.85
***Plasma homocysteine (μmol/L)***	7.8 ± 1.3	8.5 ± 3.1	7.2 ±2.0	0.41

Note: Mean values in 3 groups were compared using one way ANOVA followed by Tukey’s HSD test; p-value < 0.05 was considered significant.

Mean body weights of Sprague Dawley rats in each of the three groups (control, 30 mg betel nut per day and 60 mg betel nut per day) at the start of the experiment were nearly the same (Table 3). However, percent increases in mean body weights in the control group (group 1), group 2 (30 mg betel nut per day) and group 3 (60 mg betel nut per day) over a period of 5 weeks were 10.2%, 12.2% and 13.6%, respectively. The mean body weights (over the study period) when compared using repeated measures ANOVA, were not found to be significantly different among the 3 groups. This implies that the effect of betel nut is the same across different time intervals. The histological examination of the liver, kidney and stomach showed no remarkable change. There was no accumulation of fat in the liver of group 2 rats in spite of significantly high levels of cholesterol in this group.

**Table 3:** Mean body weights of three groups of rats receiving betel nut or placebo for 5 weeks

***Group***	***n***	***Body wt at Day 1 (gm)***	***Body wt at Day 8 (gm)***	***Body wt at Day 15 (gm)***	***Body wt at Day 22 (gm)***	***Body wt at Day 29 (gm)***	***Body wt at Day 36 (gm)***
**Mean ± SD**							
***Control (Placebo)***	12	168.5 ± 10.1	177.7 ± 10.2	182.1 ± 7.2	184.2 ± 9.4	186.7 ± 8.5	185.7 ± 9.8
***Betel nut (30 mg/day)***	12	167.0 ± 13.3	177.8 ± 14.5	184.8 ± 13.5	187.5 ± 12.6	190.1 ± 14.9	187.5 ±13.9
***Betel nut (60 mg/day)***	12	167.1 ± 10.2	177.7 ± 10.4	182.2 ±10.6	184.9 ± 10.0	189.8 ± 7.1	189.9 ± 8.5

Note: Mean body weights of rats among the 3 groups over a period of 5 weeks were compared using repeated measures ANOVA; F-statistic for the group = 0.122, p-value = 0.885; F-statistic for days = 27.529, p-value = < 0.001; F-statistic for the interaction of groups and days = 0.788, p-value = 0.578.

**Figure 1:**
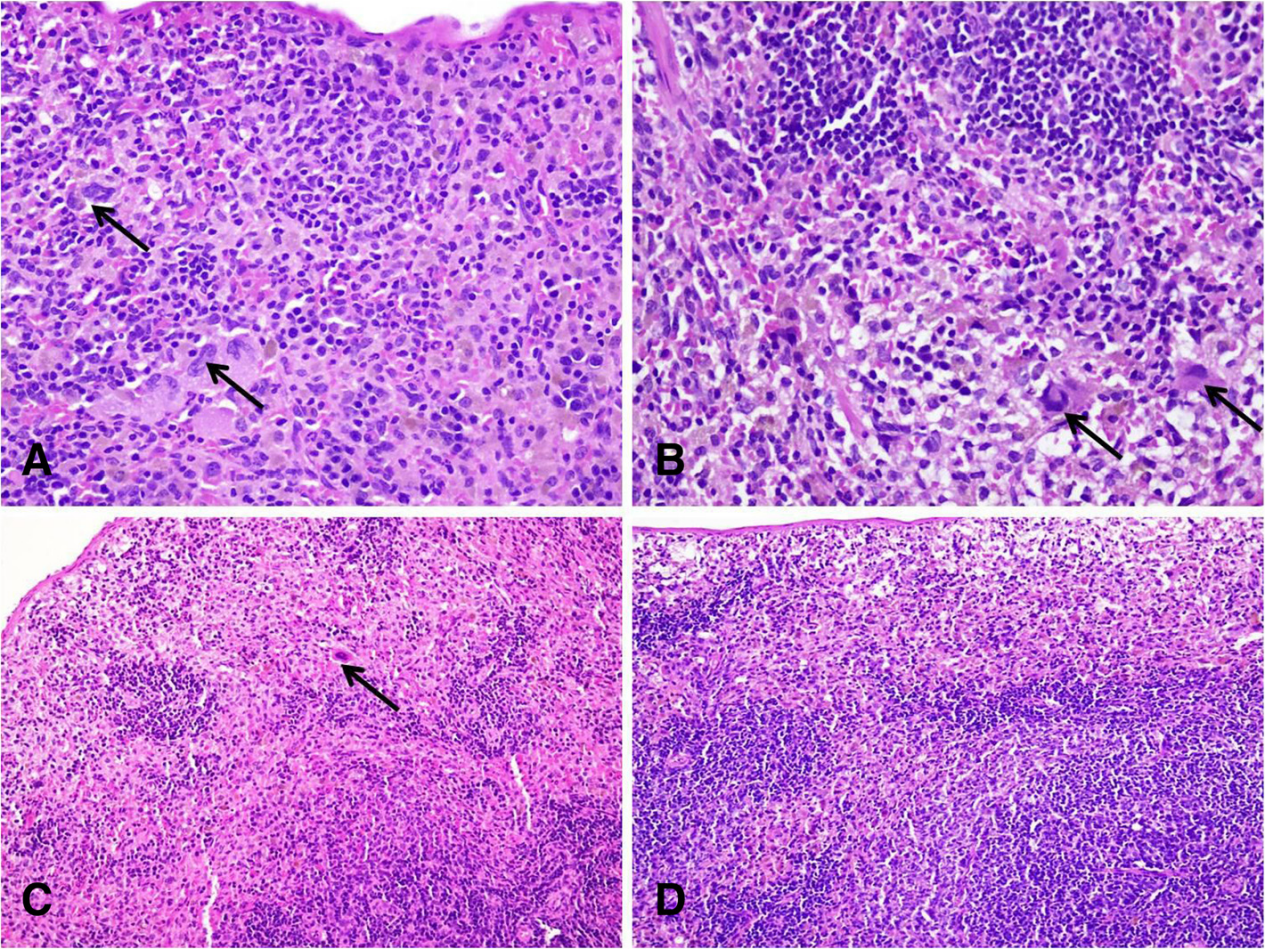
**Photomicrograph of rat spleen treated with betel nut at two different doses.** (**1A**) Section of spleen from a rat treated with 60 mg/day dose of betel nut for 5 weeks. Numerous foci of extramedullary erythropoiesis with megakaryocytes (marked by arrow). H & E, Mag: 20X. (**1B**) Photomicrograph IA at a higher magnification (Mag: 40X). (**1C**) Section of spleen from a rat treated with 30 mg/day dose of betel nut for 5 weeks. Few foci of extrameduallary hematopoiesis; megakaryote marked by arrow. H & E, Mag: 20X. (**1D**) Section of a spleen from an age-matched control rat treated with water. No extramedullary hematopoiesis is seen. H & E, Mag: 20X.

Extramedullary hematopoiesis was, however, observed in spleen in a dose dependent manner. The degree of hematopoiesis (erythropoiesis with megakaryocytes) was more pronounced in the group receiving 60 mg of betel nut per day (Figure 1A and 1B) compared to few foci in the group receiving 30 mg of betel nut per day (Figure 1C), and none in the control group treated with water (Figure 1D).

## Discussion

Nearly 600 million people all around the world indulge in betel nut chewing [2]. This habit is especially very common in 150 million South Asian populations [22]. In this population a prevalence of CVD is found in almost epidemic proportions [23]. Recent reports have indicated an association of CVD with betel nut chewing [6-8]. However, the results are conflicting about the effect of betel nut chewing on various risk factors for CVD. The differences appear to be due to the fact that different populations could be using betel nut to varying degrees, therefore, the daily rate of betel nut use would be more important than a mere “use” or “non-use” information [7]. Moreover, some of the studies reporting low serum levels of triglycerides and cholesterol were carried out using extract of betel nut in a rat model rather than a suspension of the powdered nut [24]. However, a couple of large prospective studies in Taiwan have shown a relationship between betel nut chewing and increased risk of CVD [6,7].

Our results in a rat model are novel in the sense that we observed significantly high levels of serum cholesterol at a dose of 30 mg betel nut powder per day compared to a higher dose of the betel nut. This suggests that very high consumption of betel nut might not be associated with dyslipidemia due to the inhibitory effect of high doses of betel nut on cholesterol absorption [25]. This might be one explanation why conflicting results have been reported by various investigators.

A recently published study has shown a significant association between betel nut chewing and systemic inflammation suggesting that it might be playing a role in the development of systemic diseases at a later stage [26]. Plasma NAG is a non-specific marker of inflammation. Its concentration in plasma increases in a number of inflammatory conditions [27]. Ingestion of betel nut in the present study did not produce any significant change in the concentration of plasma NAG in rats indicating that betel nut in doses upto 60 mg/day does not produce any noticeable inflammation in rats. This is further substantiated by the absence of inflammatory cells upon the histological examination of liver, kidney, spleen and stomach biopsies from various groups of rats (photograph of only spleen is shown in Figure 1). Moreover, our results pertaining to serum levels of ALT also show that liver function does not appear to be compromised by betel nut ingestion in a rodent model.

Betel nut chewing has also been found to be associated with increased risk of developing diabetes mellitus [28]. However, in the present study, ingestion of betel nut upto a dose of 60 mg/day had no effect on fasting glucose levels in a rodent model. This also indicates that rodents might be responding to betel nut in a different manner.

Mean concentrations of serum folate, vitamin B_12_ and plasma homocysteine remained similar across the 3 groups (Table 2). These findings are not in line with the reported association of folate deficiency and hyperhomocysteinemia with betel nut use in Bangladeshi human subjects [9]. The difference may be because of adequate availability of folate in the rodent diet. It has been reported that chewing betel quid or areca nut could decrease body weight in hamsters over a period of 4 months [29]. However, in the present study we did not find any significant difference in the total body weight of rats among the control group of rats, as well as those treated with betel nut over a period of 5 weeks. This shows that betel nut ingestion over a relatively short period of time has no adverse effect on the weight of growing rats.

The effect of betel nut on various body organs merits some discussion. Betel nut chewing results in exposure to its various alkaloid derivatives such as N-nitroso-compounds and polyphenols which could adversely affect various organs of the body such as stomach, liver, lung and pancreas [1]. Results of the histological examination of stomach, liver, spleen and kidney revealed no remarkable change after 5 weeks of betel nut administration except increased extramedullary hematopoiesis in spleen. Extramedullary hematopoiesis tends to be decreased in adult animals, but can increase in cases of anemia, inflammation or decreased production by bone marrow [30]. Our observation that there is a dose-dependent increase in extramedullary hematopoiesis in spleen due to betel nut suggests that higher doses of this natural product could be adversely affecting bone marrow function. However, this notion needs to be further explored.

The results of the study need to be evaluated within the context of certain limitations. Quantification of main alkaloids in the betel nut suspension for the intervention group was not carried out. This has been a limiting factor in discussing our results in the light of the main alkaloids present in the local betel nut. Moreover, the sample size in this study was relatively small. The post-hoc power of the study was calculated and found to be in the range 5% (NAG) to 99.2% (triglycerides). However, power turned out to be sufficient (85%) for the significant variable (total cholesterol concentration) in this study. We are also mindful of the fact that betel nut is normally chewed by humans. However, in this study, in order to ensure complete ingestion of the nut in two different dosages, we administered a water suspension of betel nut in the buccal cavities of rats. This indeed was a limitation; yet, our focus in this study was on determining the systemic effects of betel nut via gut absorption. Furthermore, the fact that levels of serum cholesterol in rats receiving a high dose of betel nut (60 mg/day) were lower than expected could be attributed to the inhibition of two enzymes involved in cholesterol absorption, such as pancreatic cholesterol esterase and intestinal acyl-CoA:cholesterol acyltransferase, as reported earlier in a rat model [25]. One possibility could be a dose-dependent effect; though the activity of these two enzymes was not monitored in the present study to investigate this hypothesis.

## Conclusions

The results of the investigation revealed that hypercholesterolemia was caused by a relatively small dose (30 mg/day) of betel nut in a rat model. This metabolic change was not accompanied by any significant alteration in inflammation, serum levels of HDL-cholesterol, LDL-cholesterol, triglycerides, ALT, fasting glucose, homocysteinemia and body weight. However, the findings suggest that even small doses of betel nut could have detrimental effects. Further studies on a relatively narrowed down range of dosages of betel nut would be required to find out the precise dose at which the deleterious effects of this nut begin to manifest. It is imperative that betel nut chewing must be investigated in populations which are at a greater risk of developing CVD.

## Competing interests

The authors declare that they have no competing interests.

## Authors' contribution

MPI, NM conceived and designed the study, interpreted the data and drafted the manuscript. NM and GH performed the experiments and analyzed the data. SP performed the histological studies, interpreted the data and helped in drafting the manuscript. IA performed the post-hoc power analysis of the study and helped in the interpretation of the data. All the authors read and approved the final version of the manuscript.

## Pre-publication history

The pre-publication history for this paper can be accessed here:

http://www.biomedcentral.com/1471-2261/12/94/prepub
